# Hydrophobia without Known Inoculation or Poisoning — a Case

**Published:** 1872-07-15

**Authors:** 

**Affiliations:** Mercy Hospital


### HYDROPHOBIA WITHOUT KNOWN INOCULATION OR POISONING—A CASE.

SERVICE OF PROF. DAVIS, IN MERCY HOSPITAL.

*Reported by S. — —.*

Yesterday, (Jan. 24th, 1872,) at 12 o'clock M., a patient was brought into the Hospital reported to have Hydrophobia. It was a laboring man, aged 28 years, named George Ryder. He was medium size, rather robust in appearance, and had been employed as engineer in charge of the engine in the baking house of a Mr. Bowes. It was reported that for two nights previous to his attack of sickness he had been engaged nearly all the nights at parties, dancing and engaging in the excitements usual to such occasions, and yet attending to his work during the day. It was learned from himself that he had formerly practiced masturbation freely, and had since been subject to involuntary seminal emissions. Neither himself or friends had any recollection that he had ever been bitten by dog or cat or any animal that could have been rabid. About ten days since, he had been vaccinated, which had produced an unusually large sore on his arm, still in active progress at the time of his admission. The operation of vaccination was accompanied by syncope. These are all the facts we have been able to gather concerning the history of this man prior to the attack of his present sickness.

Little more than twenty-four hours before he was brought to the hospital, his countenance became dejected, mind despondent, and slightly wandering, particularly suspicious and apprehensive. At frequent intervals he seemed to have a sense of choking or spasmodic action of the muscles of the throat, increased by attempts to drink. At times his face would be deeply suffused, his manner excited and restless, with incoherent talking, and at other times more quiet and entirely rational. These symptoms steadily increased in severity, until his spells of flushing and excitement became violent paroxysms of convulsions, characterized especially by spasmodic action of the muscles of respiration and deglutition. He soon refused all attempts to swallow liquids, and the sight or sound of water evidently aggravated all the spasmodic phenomena.

It was in this condition that he was admitted to the hospital at noon yesterday. When we saw him, one hour later, his face was still somewhat flushed; the prolabia blueish; hands and feet cool, and capillaries congested with dark blood; expression anxious and dejected; pulse 110, weak; bowels had been freely moved; urine rather scanty and high colored; moderate hyperæsthesia, especially over the abdomen; with severe spasmodic paroxysms at intervals varying from ten to forty minutes, accompanied by severe choking and mental excitement. In the interval his mind dwelt much on religious subjects and seemed to be annoyed with the idea that those around him doubted his sincerity. He could not be induced to swallow liquids, and always appeared greatly excited on the approach of water. Efforts were made to relieve his suffering and prevent the spasmodic paroxysms by hypodermic injections of sulphate of morphia, and the use of drachm doses of tincture of calabar bean, administered per rectum. Although the doses of morphia administered hypodermically were increased to two-thirds of a grain, and between four and five grains used during the fifteen hours that the patient was under treatment in the hospital, neither this nor the calabar bean, administered by the rectum, appeared to exercise any influence over the progress of the disease. During the last eight hours of the patient's suffering, D. A. K. Steele, one of the senior students who aided in taking care of him, made the following memoranda:

11 o'clock P. M. Patient is lying pretty quiet; talks as if we doubted his sincerity; prays incessantly, and asks the prayers of others.

11.30 P. M. Exhibits abnormal sensitiveness to sounds, with constant elevation and depression of eye-brows. Gave hypodermic injection of morphia.

12. Very uneasy and restless; respiration irregular; pulse small and feeble.

12.30 A. M. Continues same. Gave an injection containing one fluid drachm of tincture of calabar bean.

1 A. M. Has a slight convulsive paroxysm.

1.30 A. M. Talks rationally and is more quiet.

2 A. M. Is more restless. Gave hypodermic injection of morphia.

2.20 A. M. Had a moderate passage from the bowels and urinated somewhat spasmodically.

2.40 A. M. Had a more copious evacuation from the bowels, with much fetid gas.

3 A. M. Mind clear; sits up in an easy chair and talks much on general topics.

3.25 A. M. Had another stool.

3.45 A. M. Had been more restless and excited. Gave a hypodermic injection of morphia.

4 A. M. Had a severe spasmodic choking paroxysm, and made desperate efforts to jump out of the window.

4.30 A. M. Paroxysms continue, but lighter.

5 A. M. Shows symptoms of a severe paroxysm approaching; begs us to handcuff him —not to let him harm us; is partially delirious; asks to be shot.

5.15 A. M. Has a terrible spasm, requiring all the efforts of Dr. Waite and myself to keep him from doing himself harm. Shrieks; froths at the mouth; noise in his throat resembling the snapping of a dog; eyes glassy; circulation rapidly failing; no pulse at the wrist. Gave hypodermic injection of morphia.

6 A. M. Continues to have spasms every few minutes. Begs to be put out of his misery.

6.30 A. M. Paroxysms more violent.

6.45 A. M. Fast sinking.

7 A. M. Death, apparently from syncope.

Such is the brief history of this case. The patient was seen by two or three intelligent physicians before he was brought to the hospital, and by several members of our college faculty, including Professor Andrews, while here, and all agree that the symptoms throughout were those of well-marked hydrophobia. It is reasonably certain, however, that they were not induced by the bite of any rabid animal. Was there some peculiar animal poison absorbed from the large and unhealthy vaccine sore on his arm? Or did the excitement of dancing parties at night and work during the day, coöperating with the disturbing influence of the vaccine virus establish the irritation of the nervous centers on which the violent symptoms depended? Can hydrophobia be induced without the agency of a specific virus derived from a rabid animal? These are interesting questions suggested by the case under consideration, but which are not easily answered. Some have claimed that hydrophobia is simply a peculiar affection of the cerebro-spinal nervous centers, and have expressed doubts about the existence of any such poison as rabies. The facts pointing to the existence and destructive influence of such a poison, are too numerous, however, to admit of reasonable doubt. And yet we are compelled to admit that cases presenting all the phenomena of hydrophobia have occurred that could not be traced to the introduction of any such poison. Such is, indeed, the case before us.

A *post mortem* examination having been made in this case, we present you the brain and its membranes, the medulla oblongata and two inches of the spinal cord. There is no noticeable effusion of any kind, either upon the surface or into the ventricles of the brain. Neither is there more than slight appearances of increased vascularity in the pia-mater covering the upper, anterior, and lateral parts of the hemispheres; but you see that portion of the membrane covering all the base of the brain most intensely and beautifully injected with red blood; and as we separate the convolutions of the base of the middle lobes, a slight degree of adhesion from the exudation of plastic lymph is discernible; and in slicing off portions of the convolutions, the gray substance exhibits distinct increased vascularity. These evidences of inflammatory action and hyperæmia are most marked over the base of the right hemisphere, dipping deeply into the sulcus leading to the bottom of the right ventricle and over the pons verolii and medulla oblongata. It is to this local inflammatory condition of the membrane and base of the brain, including the important nervous centers of respiration and muscular motion, that we must attribute the sickness and death of the patient. And we are inclined to think the true cause of such local inflammation is suggested by the second question of the three which we stated a few moments since. We have been told that one physician who saw the patient in the incipient stage of his attack, strongly recommended general bleeding, and judging from the *post mortem* appearances, it is highly probable that such practice would have been beneficial at that stage. But the functions of the base of the brain and medulla oblongata, are so intimately connected with the maintenance of respiration and circulation, that acute attacks of disease involving those parts will always result in a high ratio of mortality, regardless of treatment.

